# Supplementary material for: Using Telehealth to Guarantee the Continuity of Rehabilitation during the COVID-19 Pandemic: A Systematic Review
Source: Int J Environ Res Public Health. 2022 Aug 19;19(16):10325. doi: 10.3390/ijerph191610325 (PMC9408792; doi:10.3390/ijerph191610325)
Supplement: Supplementary file 1 [file ijerph-19-10325-s001.zip › ijerph-1789487-supplementary.pdf]

Supplementary Table S1. Search strategies used for each database

Database	Search Query	Filters and Limits Applied	Articles Returned
Pubmed	(telemedicine OR telemetry OR telerehabilitation OR tele-rehabilitation OR telerehab OR telephysiotherapy OR "remote rehabilitation" OR "remote physiotherapy" telehealth OR tele-health OR telehomecare OR tele-homecare OR telecoaching OR tele-coaching OR telecommunication* OR videoconference OR video-conferenc* OR videoconsultation OR video-consultation OR telestroke OR teleconferenc* OR tele-conferenc* OR teleconsultation OR tele-consultation OR telecare OR ehealth OR e-health) AND (COVID OR COVID-19 OR coronavirus-19 OR "coronavirus disease 2019" OR SARS-CoV-2) NOT protocol<br><br>(((telerehabilitation[MeSH Terms]) OR (telerehab[MeSH Terms]) OR (telephysiotherapy[MeSH Terms]) OR (remote rehabilitation[MeSH Terms]) OR (remote physiotherapy[MeSH Terms]) OR (telehealth[MeSH Terms]) OR (telehomecare[MeSH Terms]) OR (telecoaching[MeSH Terms]) OR (videoconference[MeSH Terms]) OR (videoconsultation[MeSH Terms]) OR (telecare[MeSH Terms]) OR (ehealth[MeSH Terms])) AND ((COVID[MeSH Terms]) OR (coronavirus disease 2019[MeSH Terms]) OR (SARS-CoV-2[MeSH Terms])) NOT protocol)	Published up to May 31st 2022,<br><br>Limited to: Clinical study, Clinical trial, Observational Study, RCT, pragmatic RCT	265<br><br>+<br><br>194
PEDro	Telerehabilitation AND Covid-19;<br>Telephysiotherapy AND Covid-19;<br>Telehealth AND Covid-19.	Clinical Trials	6
Cochrane Library	(telemedicine OR telemetry OR telerehabilitation OR tele-rehabilitation OR telerehab OR telephysiotherapy OR “remote rehabilitation” OR “remote physiotherapy” OR telehealth OR tele-health OR telehomecare OR tele-homecare OR telecoaching OR tele-coaching OR telecommunication* OR videoconference OR video-conferenc* OR videoconsultation OR video-consultation OR telestroke OR teleconference* OR tele-conference* OR teleconsultation OR tele-consultation OR telecare OR ehealth OR e-health) AND (COVID OR COVID-19 OR coronavirus-19 OR "coronavirus disease 2019” OR SARS-CoV-2) NOT (protocol) in “Title Abstract Keyword” (Word variations have been searched)	Clinical Trials with Cochrane Library publication date up to 31st May 2022	464
Web of Science	(telemedicine OR telemetry OR telerehabilitation OR tele-rehabilitation OR telerehab OR telephysiotherapy OR "remote rehabilitation" OR "remote physiotherapy" telehealth OR tele-health OR telehomecare OR tele-homecare OR telecoaching OR tele-coaching OR telecommunication* OR videoconference OR video-conferenc* OR videoconsultation OR video-consultation OR telestroke OR teleconferenc* OR tele-conferenc* OR teleconsultation OR tele-consultation OR telecare OR ehealth OR e-health) AND (COVID OR COVID-19 OR coronavirus-19 OR "coronavirus disease 2019" OR SARS-CoV-2) NOT protocol	Document Types: Articles;<br><br>Categories: Rehabilitation	32

Supplementary Table S2: Quality Assessment of Randomized Controlled Trials [23-28]

PEDro Scale	(Eligibility criteria specified)	Random allocation	Concealed Allocation	Baseline Comparability:	Blind Subjects	Blind Therapists	Blind Assessors	Follow-up >85% of subjects	Intention-to-treat analysis	Between-group Statistics	Point measures & Variability	Total Score
Batalik et al. (2021)	(No)	Yes	No	Yes	No	No	No	Yes	Yes	Yes	Yes	6/10
Gonzalez-Gerez et al. (2021)	(Yes)	Yes	No	Yes	Yes	No	Yes	Yes	Yes	Yes	Yes	8/10
Hernando-Garijo et al. (2021)	(Yes)	Yes	No	Yes	No	No	Yes	Yes	Yes	Yes	Yes	7/10
Li et al. (2021)	(Yes)	Yes	Yes	Yes	No	No	Yes	Yes	Yes	Yes	Yes	8/10
Ozturk et al. (2022)	(Yes)	Yes	No	Yes	No	No	No	Yes	No	Yes	Yes	5/10
Rodríguez-Blanco et al. (2021)	(Yes)	Yes	No	Yes	Yes	No	Yes	Yes	Yes	Yes	Yes	8/10

Supplementary Table S3: Quality Assessment of Observational Studies [22,29-37]

	STROBE Checklist	Cancino-López et al. (2021)	De Marchi et al., (2020)	Lamberti est al., (2021)	Milani et al. (2021)	Negrini et al., (2020)	Oprandi et al., (2021)	Patel et al. (2021)	Romano et al. (2021)	Sakai et al. (2020)	Werneke et al. (2021)
	Title & abstract (Study design & summary)	Yes	Yes	Yes	Yes	Yes	Yes	Yes	Yes	Yes	Yes
Intro	Background	Yes	Yes	Yes	Yes	Yes	Yes	Yes	Yes	Yes	Yes
	Objectives	Yes	Yes	Yes	Yes	Yes	Yes	Yes	Yes	Yes	Yes
Methods	Study design (key elements)	Yes	Yes	Yes	Yes	Yes	Yes		Yes	Yes	Yes
	Setting	Yes	Yes	Yes	Yes	Yes	Yes		Yes	Yes	Yes
	Participants	Yes	Yes	Yes	Yes	Yes	Yes	Yes	Yes	Yes	Yes
	Variables	Yes	Yes	Yes	Yes	Yes	Yes	Yes	Yes	Yes	Yes
	Data sources/ measurement	Yes	Yes	Yes	Yes	Yes	Yes	Yes	Yes	Yes	Yes
	Bias										
	Study size			Yes				Yes	Yes		
	Quantitative variables	Yes	Yes	Yes	Yes		Yes	Yes	Yes	Yes	Yes
	Statistical methods	Yes	Yes	Yes	Yes	Yes	Yes		Yes	Yes	Yes
Results	Participants	Yes	Yes	Yes	Yes		Yes	Yes	Yes	Yes	Yes
	Descriptive data	Yes	Yes	Yes	Yes	Yes	Yes	Yes	Yes	Yes	Yes
	Outcome data	Yes	Yes	Yes	Yes	Yes	Yes	Yes	Yes	Yes	Yes
	Main results	Yes	Yes	Yes	Yes	Yes	Yes	Yes	Yes	Yes	Yes

	**Other analyses**			Yes	Yes						Yes
	**Key results**	Yes	Yes	Yes	Yes	Yes	Yes	Yes	Yes	Yes	Yes
	**Limitations**	Yes	Yes	Yes	Yes	Yes	Yes	Yes	Yes	Yes	Yes
**Discussion**	**Interpretation**	Yes	Yes	Yes	Yes	Yes	Yes	Yes	Yes	Yes	Yes
	**Generalisability**	Yes	Yes	Yes	Yes	Yes	Yes	Yes	Yes	Yes	Yes
	**Funding**		Yes	Yes			Yes		Yes		Yes
	**Total Score:**	18/22	19/22	21/22	19/22	16/22	19/22	16/22	20/22	18/22	20/22

Supplementary Table S4: Quality Assessment of Feasibility and Pilot Studies [38-41]

	CONSORT Checklist	Lowe et al. (2021)	Martin et al., (2021)	Nakayama et al., (2020)	Tanguay et al., (2021)
	Title & abstract (Study design & summary)	Yes	Yes	Yes	Yes
Intro	Background & Objectives	Yes	Yes	Yes	Yes
Methods	Trial Design	Yes	Yes	Yes	Yes
	Participants	Yes	Yes	Yes	Yes
	Interventions	Yes	Yes	Yes	Yes
	Outcomes	Yes	Yes	Yes	Yes
	Sample Size				
	Sequence Generation				
	Allocation Concealment Mechanism				
	Implementation				
	Blinding				
	Statistical Methods		Yes	Yes	Yes
Results	Participants Flow	Yes	Yes	Yes	Yes
	Recruitment	Yes	Yes	Yes	Yes
	Baseline Data		Yes	Yes	Yes
	Numbers Analyzed	Yes	Yes	Yes	Yes
	Outcomes & Estimations		Yes	Yes	Yes
	Ancillary Analyses		Yes		
	Harms				
Discussion	Limitations	Yes	Yes	Yes	Yes
	Generalisability	Yes	Yes	Yes	Yes
	Interpretation	Yes	Yes	Yes	Yes
Other Info	Registration	Yes			
	Protocol	Yes			

	**Funding**	Yes	Yes	Yes	
	**Total Score:**	17/25	17/25	16/25	15/25
